# A Report of a Rare Case of Meniscus in the Humeroradial Joint Causing Elbow Snapping, Treated Arthroscopically

**DOI:** 10.7759/cureus.97340

**Published:** 2025-11-20

**Authors:** Christos Lyrtzis, Christos Koukos, Georgios Trikoilis, George Paraskevas

**Affiliations:** 1 Department of Anatomy and Surgical Anatomy, Faculty of Health Sciences, School of Medicine, Aristotle University of Thessaloniki, Thessaloniki, GRC; 2 Department of Orthopedics, Sports Trauma and Pain Institute, Thessaloniki, GRC

**Keywords:** arthroscopy, brachioradial meniscus, elbow, snapping elbow, synovial fold

## Abstract

Lateral-sided elbow snapping is a rare clinical condition characterized by a painful, audible click during flexion and extension of the forearm over the lateral aspect of the elbow joint. It is typically attributed to intra-articular pathology such as inflammation or thickened synovial plicae, redundant or torn bundles of the annular ligament, and anatomical variations, such as the existence of a meniscus inside the joint cavity. We report a rare case in which the underlying cause was a brachioradial meniscus within the radiohumeral joint. Magnetic resonance imaging (MRI) suggested the presence of an interposed meniscus-like structure, which was confirmed during diagnostic arthroscopy. Arthroscopic ablation of the interposed tissue was performed, resulting in complete resolution of symptoms and restoration of full function. The brachioradial meniscus is an uncommon anatomical variant, with only a few cases previously documented. Although exceptionally rare, it should be considered in the differential diagnosis of painful lateral elbow snapping to avoid diagnostic oversight and inappropriate management. Early recognition and arthroscopic management can ensure excellent functional outcomes and prevent secondary chondral injury.

## Introduction

Snapping elbow is a pathological condition characterized by a clicking or snapping sensation typically occurring during elbow flexion with the forearm in pronation. This is a relatively rare occurrence. Lateral snapping over the radial head is typically associated with intra-articular pathology: the cause has been identified as a synovial fold or a loose annular ligament that becomes temporarily trapped in the radiocapitellar joint during motion [1]. Another potential cause is the existence of an interposed meniscus between the articular surfaces of the humeroradial joint. This fibroadipose structure, normally found in the knee joint, can become temporarily trapped inside the humeroradial joint when interposed between the humeral capitellum and the fovea of the radial head, resulting in painful snapping. Lateral snapping of the elbow joint caused by a meniscus is a very rare clinical condition, first reported by Huang et al. [2]. After that, another two cases were reported by Kang and Kim [3], while the meniscus was also found in the humeroradial joint of a 15-month-old infant by Fabié et al. [4]. We report an uncommon case of painful lateral-sided snapping of the elbow joint caused by this very rare anatomical abnormality, treated arthroscopically with ablation of the interposed structure.

## Case presentation

**Figure 1:**
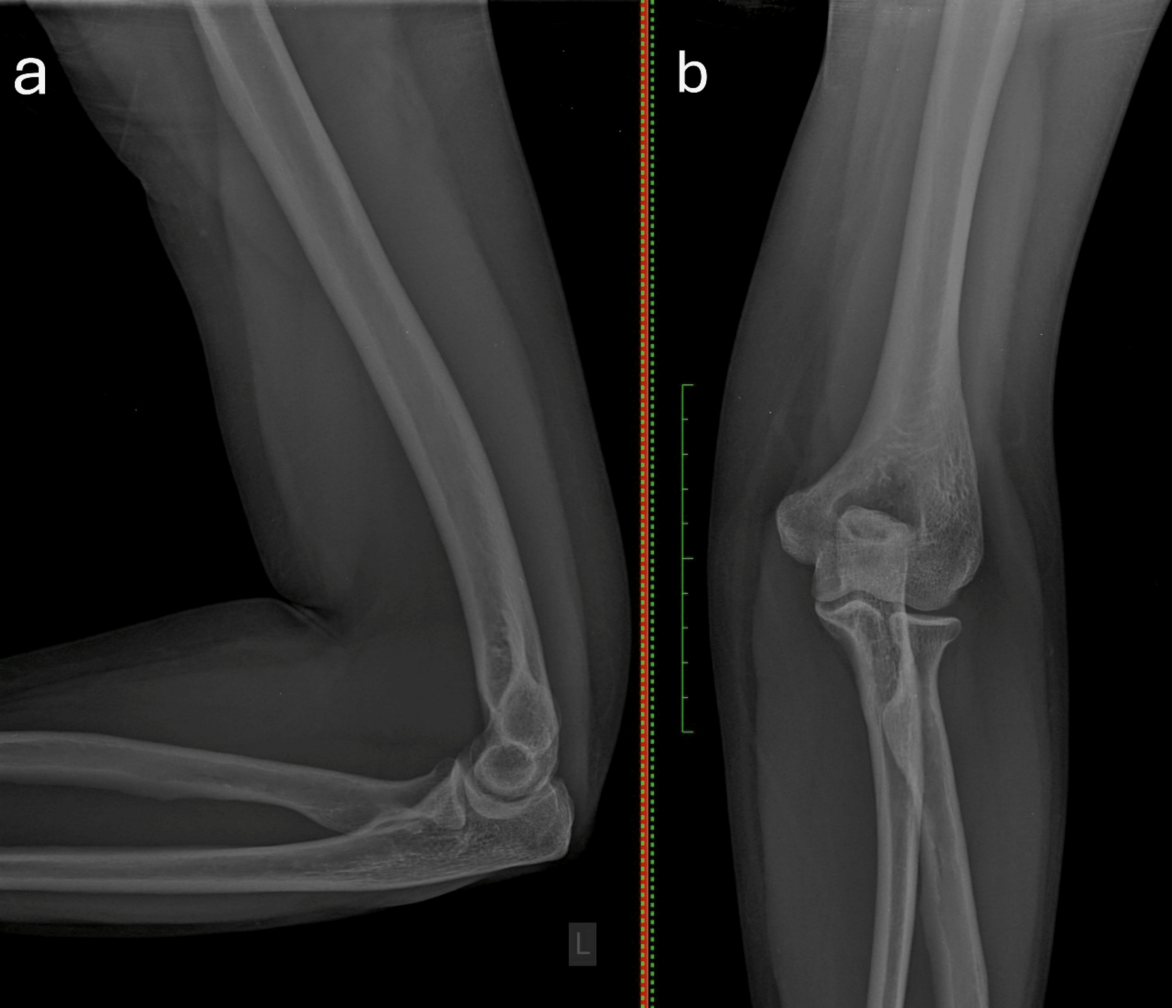
Plain radiographs of the left elbow: (a) lateral view and (b) anteroposterior view, demonstrating normal bony alignment and joint spaces. No pathological findings were observed.

A 22-year-old right-handed female volleyball player presented to our clinic with a two-year history of pain and snapping over the lateral aspect of the left elbow, occurring predominantly during forearm flexion and extension, with the forearm in full pronation. The patient reported that pain and discomfort interfered with sports and routine activities. There was no reported history of prior elbow trauma. During clinical examination, no deficit in the range of motion (ROM) of the elbow was observed. The ROM examination results of the elbow were within normal limits, with extension-flexion measured at 0°/140° and forearm supination-pronation measured at 80°/0°/80°. There was tenderness on the anterior soft spot. Elbow flexion performed with the forearm in full pronation reproducibly elicited the snapping sound throughout the 70°-120° range of flexion/extension. Additionally, a slightly positive posterior drawer was shown, indicating a minor posterior subluxation of the radial head. Radiographs of the affected elbow showed no abnormal findings (Figure 1).

**Figure 2:**
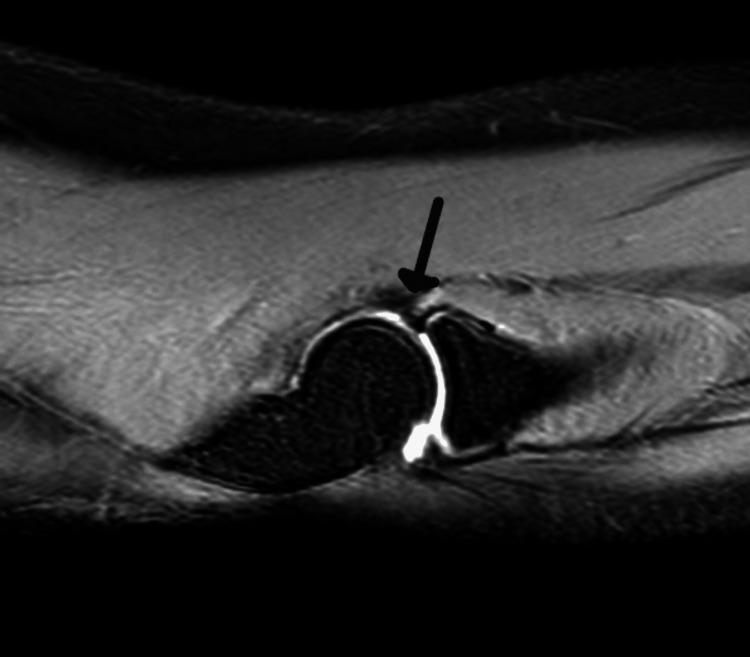
Magnetic resonance imaging (MRI) demonstrating a triangular-shaped, low-signal-intensity structure (arrow) interposed between the radial head and humeral capitulum, located anterior to the radiocapitellar joint. This finding was suggestive of a meniscus.

Due to the persistence of painful snapping, magnetic resonance imaging (MRI) of the affected elbow was performed. The imaging findings implied the presence of a meniscus within the elbow joint (Figure 2).

**Figure 3:**
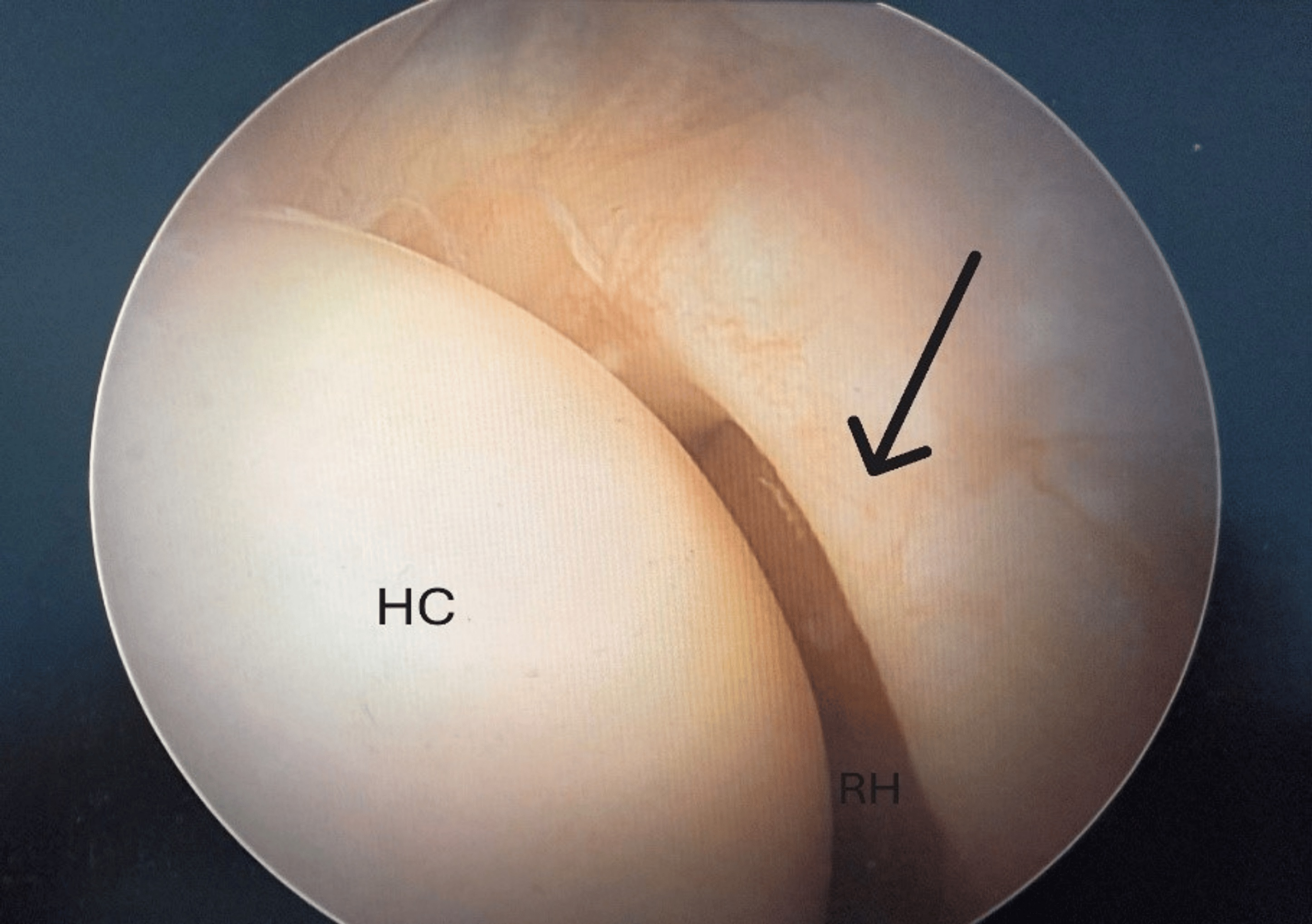
Arthroscopic view of the radiocapitellar joint of the patient revealed a white, semilunar-shaped meniscus with a free medial edge (arrow), interposed between the humeral capitellum (HC) and the radial head (RH).

Subsequently, diagnostic arthroscopy was undertaken, which confirmed the existence of a white semilunar-shaped tissue that had a free inner edge, a meniscus (Figure 3).

**Figure 4:**
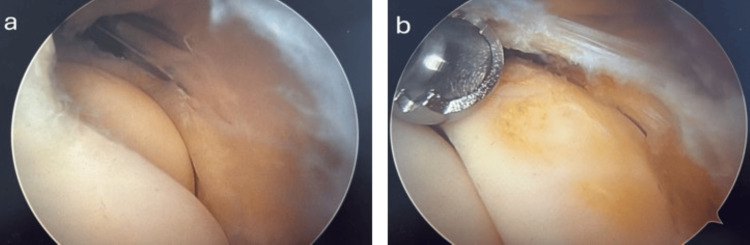
Arthroscopic images of the radiocapitellar joint obtained during (a) and after ablation (b) of the interposed meniscus, while preserving the annular ligament without removing it. No residual tissue was observed between the radial head and the humeral capitellum following ablation.

This semilunar-shaped structure was located anterolaterally to the humeroradial joint, with its free inner edge interposed within the joint. We removed the meniscus arthroscopically via ablation, preserving the annular ligament without resection (Figure 4).

Postoperatively, the patient reported resolution of symptoms and achieved a return to routine activities after a month. Moreover, she returned to her previous sports activities at the same level after three months. No complications were observed.

## Discussion

The elbow is a complex hinge joint involving three separate articulations sharing a common synovial cavity. The ulnohumeral, radiohumeral, and proximal radioulnar joints allow combinations of flexion and extension of the elbow and pronation and supination of the forearm. The joint is stabilized by the medial collateral ligament (MCL) and the lateral collateral ligament (LCL). The MCL consists of the anterior oblique ligament (AOL), the posterior oblique ligament (POL), and the transverse ligament (TL), also known as Cooper's ligament, while the LCL consists of the lateral radial collateral ligament (LRCL), the annular ligament (AL), and the lateral ulnar collateral ligament (LUCL) [5]. The anatomical stabilizers of the elbow joint are categorized as primary and secondary. The primary stabilizers consist of the MCL, the LUCL, and the osseous components of the ulnohumeral articulation, namely the olecranon, trochlea, and coronoid process. The secondary stabilizers include the joint capsule; the common flexor origin (CFO) and common extensor origin (CEO); and the osseous components of the radiohumeral joint, specifically the fovea of the radial head and the capitellum of the humerus. Additionally, the anconeus muscle may serve as a dynamic stabilizer of the elbow joint [6]. In healthy individuals, flexion ranges between 130° and 154°, extension from -6° to 11°, pronation from 75° to 85°, and supination from 80° to 104° [7]. Limitation or pain in elbow mobility adversely affects functional tasks, including lifting, grasping, and overhead reaching, and may contribute to considerable functional impairment and reduced quality of life.

This study showed that one possible cause of painful snapping of the elbow can be the interposition of a meniscus in the humeroradial joint. An interposed meniscus in the humeroradial joint is a rare anatomical abnormality that is not typically included among the intra-articular structures of the elbow joint. According to Mercer and Bogduk, the normal intra-articular components comprise the fat pads, located opposite the olecranon fossa, radial fossa, and coronoid fossa, as well as anteriorly between the radial head and the trochlear notch of the ulna, along with connective tissue rims extending around the anterior margins of the radius, the ulna, or both. Additionally, fibroadipose structures are present adjacent to the medial and lateral aspects of the joint capsule, occupying the non-articular surfaces of the trochlear notch [8]. This meniscus is white in color, triangular or semilunar in shape, and has a fibrocartilaginous texture almost identical to that of the knee meniscus. It contains collagen fibers that run longitudinally near the surface and are arranged peripherally in the deeper layers, while it consists of small, atypical cells resembling chondrocytes and fibroblasts, and demonstrates light vascularization [4]. It is not surrounded by a membrane. The meniscus has a broad outer portion resembling a bow tie at its periphery and is located on the anterolateral surface of the humeroradial joint [2]. The structure has a free medial edge, while its base is attached to the synovial membrane near the annular ligament [3]. Its dimensions have been reported as 17 × 7 × 3 mm by Kang and Kim, 15 × 5 × 3 mm by Huang et al., and in a newborn, Fabié et al. reported an area of 2 cm², with a thickness ranging from 2 to 8 mm [2,3,4]. In our case, the meniscus was approximately 10 × 5 × 3 mm, as can be evaluated by the dimensions of the tools used during arthroscopy. The exact size of the meniscus could not possibly be measured during the arthroscopy because of the small size of the elbow joint and the method of arthroscopic ablation we used to remove it.

The snapping sound is caused by the meniscus temporarily slipping out of the joint when the forearm is flexed to approximately 120°, and passively returning to interpose between the condyle and the radial head fossa when the forearm is extended to approximately 70°. This mechanism explains the consistently reproducible snapping sound throughout the 70°-120° range of flexion/extension. Pain is caused by increased pressure on adjacent nerves due to the meniscus during elbow flexion and extension. Chronic irritation of the meniscus from repeated joint stress may lead to an inflammatory synovial reaction at its attachment site, as well as chondromalacia of the radial head and humeral condyle [9]. In our case, the meniscus located anterolateral to the humeroradial joint was slightly displaced the radial head posteriorly, resulting in minor posterior subluxation and mild joint instability. Nerve irritation caused by the snapping and the minor posterior subluxation of the radial head may account for the anterior soft spot tenderness observed in our patient. 

Painful snapping of the elbow joint may occur near either the lateral or medial epicondyle. Lateral snapping is associated with intra-articular pathology, including interposed thickened or inflamed synovial plicae [10], redundant or torn bundles of the annular ligament [11], or the presence of a meniscus within the humeroradial joint. According to Kotsapas et al., snapping and pain over the lateral aspect of the elbow may be observed in cases of posterolateral rotatory instability (PLRI). These symptoms are often accompanied by a reduced range of motion, mechanical locking, and a sense of apprehension during elbow movement. PLRI typically results from high-energy trauma, such as elbow dislocation or a fall onto an outstretched hand, but may also arise due to repetitive microtrauma associated with sports activities or repetitive daily tasks [6]. The key differentiating feature between PLRI and the presented case lies in the elbow’s ROM. In PLRI, ROM is restricted, whereas in cases involving an interposed meniscus, it typically remains within normal limits. In contrast, medial snapping is attributed to extra-articular causes, such as subluxation of the ulnar nerve or dislocation of a portion of the triceps tendon [1]. Therefore, the initial step in evaluating a patient with a snapping elbow is to determine whether the snapping occurs laterally or medially, as this distinction is essential for differentiating between intra-articular and extra-articular pathology. In cases of lateral snapping, such as the one presented here, the interposed tissue inside the elbow joint may be identified using imaging modalities such as conventional arthrography, magnetic resonance (MR) arthrography, high-resolution magnetic resonance imaging (MRI), or diagnostic arthroscopy. It is important to note that standard MRI and plain radiographs often appear normal, which may lead to misdiagnosis or confusion with more common conditions, such as lateral epicondylitis [3]. Hence, we consider diagnostic arthroscopy to be the most valuable tool for accurately identifying the cause of lateral elbow snapping, as it allows for direct inspection of the interposed intra-articular tissues. Differential diagnosis between inflamed or thickened synovial plicae, redundant or torn bundles of the annular ligament, and an interposed meniscus is based on their distinct histological characteristics, which serve as key diagnostic features. Synovial plicae are enclosed by a membrane, in contrast to the meniscus, and are composed of fibrous connective tissue and adipose tissue, but lack chondrocytes, which are present in the meniscus, while they exhibit more pronounced vascularization compared to the meniscus [4]. In contrast, the annular ligament bundles consist exclusively of fibrous connective tissue, without the adipose or cartilaginous elements observed in the other two structures. Accurate histological differentiation between synovial plicae and the meniscus is critical for establishing the correct diagnosis and guiding appropriate treatment. The condition is typically addressed through arthroscopic resection or ablation of the interposed tissue [12]. Early surgical intervention is recommended to prevent progressive degenerative changes in the articular cartilage associated with chronic snapping [13,14].

## Conclusions

This study presents a case of lateral elbow snapping caused by a meniscus within the synovial cavity of the elbow joint, a rare anatomical variant. Although uncommon, awareness and consideration of this entity as a potential cause of lateral elbow pain are essential to avoid misdiagnosis. Diagnostic arthroscopy was performed to confirm the presence of a meniscus within the humeroradial joint, and arthroscopic ablation was subsequently used to remove it while preserving the annular ligament. Consequently, arthroscopic evaluation plays a crucial role in both confirming the diagnosis and facilitating effective surgical management when the symptoms persist.
